# Principal Components Analysis of Atopy-Related Traits in a Random Sample of Children

**DOI:** 10.5402/2011/170989

**Published:** 2011-06-15

**Authors:** Simon Francis Thomsen, Vibeke Backer

**Affiliations:** Department of Respiratory Medicine, Bispebjerg Hospital, 2400 Copenhagen, Denmark

## Abstract

*Aim*. To study the relationship between atopy-related traits in a random sample of children. *Methods*. A total of 1007 randomly selected children, 7–17 years of age, from Copenhagen, Denmark were studied. The children were interviewed about symptoms of atopic diseases, and skin test reactivity, serum total IgE, lung function, and airway responsiveness were measured. Principal components analysis was performed in order to examine the relationship between the different traits. *Results*. Most of the studied traits were significantly correlated. A three-component solution explained about 55% of the variation in the observed traits. The first component loaded most strongly on hay fever, serum total IgE, skin test reactivity and sensitisation to grass, cat and house dust mite allergen; the second factor was most associated with asthma, airway obstruction, and airway hyperresponsiveness, whereas the third factor corresponded most strongly to atopic dermatitis. There was some indication of cross-relations between the three components with respect to serum total IgE. *Conclusion*. Asthma, hay fever, and atopic dermatitis are characterised by different sets of biomarkers suggestive of a high degree of heterogeneity within the atopic syndrome.

## 1. Introduction

Asthma, hay fever, and atopic dermatitis often cooccur, and this can be attributed both to shared genetic and environmental risk factors [1]. Furthermore, different biomarkers for atopic disease, such as serum IgE, lung function, airway responsiveness, airway inflammation, atopic sensitisation, and serum eosinophilia, have been shown to correlate, also in part because of a shared aetiological basis [2]. However, the correlations between both the atopic diseases and their intermediate phenotypes are incomplete. Notably, asthma and airway hyperresponsiveness (AHR) are closely linked, but far from all asthmatics exhibit AHR [3]. Furthermore, airway obstruction and low lung function are inconstant features of asthma. Atopic sensitisation is closely associated both with hay fever, asthma, and atopic dermatitis, but this association seems to be stronger in children than in adults [4]. Sensitisation to specific allergens is seen both in asthma and hay fever but with sensitisation to indoor allergens like house dust mite being more closely linked to asthma, whereas hay fever more often is characterised by sensitisation to outdoor allergens like, for example, grass pollen [4]. Studying the relationship between atopic diseases and their intermediate phenotypes can help in elucidating novel pathways towards which different treatments can be targeted. We performed a principal components analysis of a series of clinical and intermediate indicators of atopic disease in a general population of children. 

## 2. Methods

### 2.1. Population

Two different random samples of children from Copenhagen, Denmark were studied. The first sample was examined in 1986 [5] and the second in 2001 [6]. All subjects were randomly ascertained through The Civil Registration System and had a mean age of 12 years, age-range 7–17 years. In 1986 and 2001, a total of 1000 and 1500 subjects, respectively, were identified. However, due to immigration only 983 and 1440 subjects, respectively, were eligible for the studies in 1986 and 2001. A total of 527 (53.6%) participated in 1986 whereas 480 (33.3%) participated in 2001. Due to the low participation rates, telephone interviews were conducted among 100 and 116 randomly selected families from the group of nonrespondents in 1986 and 2001, respectively. For every household, the interview was first performed with the child, and subsequently a parent was consulted to reach consensus. The subjects interviewed by telephone did not differ significantly from the subjects who were clinically examined with respect to sex, age, and predisposition to atopic disease, but on both occasions there were significantly fewer children with symptoms of allergy among the group of nonrespondents. Only data for subjects who participated in the clinical examination were included in the present analysis. The local Scientific Ethics Committee approved the study, and informed consent was obtained from all participating subjects and their parents. 

### 2.2. Clinical Interview

All participants were interviewed about atopic diseases. Subjects were considered to have asthma, hay fever, and atopic dermatitis if they responded affirmatively to a series of questions adopted from the American Thoracic Society [7]. For details, see [6, 8, 9]. 

### 2.3. Skin Prick Test and Measurement of Serum Total IgE

Skin prick tests (SPTs) were performed using standard dilutions of nine common aeroallergens. The allergens used were birch, grass, mugwort, horse, dog, cat, house dust mite (HDM) (*Dermatophagoides pteronyssinus*), and mould (*Alternaria iridis* and *Cladosporium herbarium*). The concentrations of allergen were 100.000 BU/mL (Phazet system; Pharmacia, Denmark) in 1986 and 10 HEP (Soluprick SQ system; ALK Albelló, Denmark) in 2001. Reactions were read after 15 minutes. A positive result was defined as a positive reaction to at least one of the allergens, and a reaction was considered positive if the mean wheal diameter was at least 3 mm. The participants were requested to discontinue medications that contained antihistamines at least 3 days before skin testing.

 Levels of serum total IgE were measured with a paper radio immunosorbent test (PRIST, Pharmacia, Copenhagen, Denmark) in 1986 and with an enzyme-linked immunosorbent assay (ELISA) on Immulite 2500 (DPC, New York, USA) in 2001. Results were expressed as KIU/L. 

### 2.4. Lung Function and Bronchial Responsiveness Test

The preprovocation values of forced expiratory volume in 1 second (FEV_1_) and forced vital capacity (FVC) were measured and the ratio FEV_1_/FVC was calculated. The methods by Cockcroft et al. and Yan et al. were used for measuring airway responsiveness to inhaled histamine in 1986 and 2001, respectively [10, 11]. According to the Cockcroft method, a Wright nebulizer delivered the histamine and the subjects inhaled by normal tidal volume breathing. Nine concentrations of histamine were used, from 0 (saline) to 8 mg/mL, and the test was terminated when the maximum concentration was reached or when a drop in FEV_1_ of more than 20% was observed—the provocative concentration (PC_20_). AHR was defined as a PC_20_ of 8 mg/mL. According to the method by Yan, each aerosol was inhaled starting with saline and followed by increasing doses of histamine until a cumulative dose of 7.8 *μ*mol had been reached. The test was terminated when the maximum concentration had been reached or when a 20% decline in FEV_1_ had occurred before the end of the dosing regimen. For all subjects experiencing at least a 20% decline in FEV_1_, the concentration causing a 20% fall in FEV_1 _(PD_20_) was calculated. AHR was defined as a PD_20_ below 3.9 *μ*mol. 

### 2.5. Statistical Analysis

The following variables were included in the analysis: asthma, hay fever, atopic dermatitis, FEV_1_/FVC, AHR, serum total IgE, positive skin prick test and sensitisation to grass, cat, and HDM allergen. Principal components analysis was used to examine the correlational structure of the data. For the optimal solution we used varimax rotation with Kaiser normalisation. Only components with eigenvalues above 1.0 were retained in the solution. The data were analysed with the statistical package SPSS 17.0 (SPSS Inc Chicago, IL, USA). 

## 3. Results

The prevalence of asthma, hay fever, and atopic dermatitis was 7.1, 17.3, and 22.1%, respectively. The overall rate of atopic sensitisation was 21.8%, whereas the prevalence of AHR was 11.6% (Table 1). Significant correlations were observed among most of the traits (test of sphericity, *P* < .001). Particularly, positive skin prick test and serum total IgE correlated well with all other traits (except FEV_1_/FVC). Asthma was most strongly correlated with AHR (*r* = 0.38), whereas hay fever correlated most with positive skin prick test (*r* = 0.46) and grass allergen (*r* = 0.43). Cat allergen was significantly correlated with all three atopic diseases (asthma (*r* = 0.25), hay fever (*r* = 0.34), and atopic dermatitis (*r* = 0.18)). FEV_1_/FVC was most strongly associated with AHR (*r* = −0.18).

**Figure 1:**
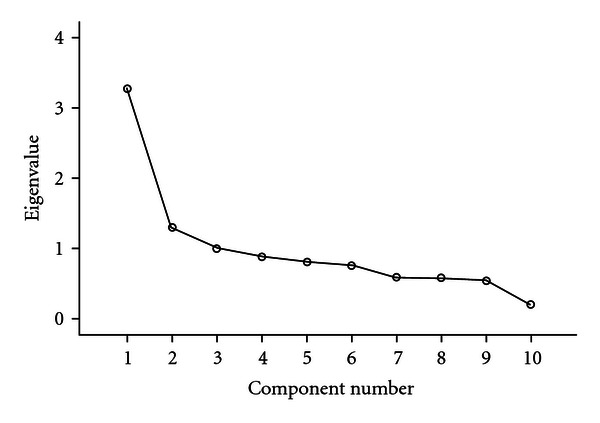
Scree plot showing the results of a principal components analysis of atopy-related traits studied in a random sample of 1007 children, 7–17 years of age.

 A three-component solution explained about 55% of the variation in the observed traits (Figure 1). The first component loaded most strongly on hay fever (*r* = 0.66), serum total IgE (*r* = 0.50), skin test reactivity (*r* = 0.89) and sensitisation to grass (*r* = 0.69), cat (*r* = 0.65), and house dust mite (*r* = 0.71) allergen; the second factor was most associated with asthma (*r* = 0.67), airway obstruction (*r* = −0.62), and airway hyperresponsiveness (*r* = 0.74), whereas the third factor corresponded most strongly to atopic dermatitis (*r* = 0.90). There was some indication of cross-relations between the three components in relation to serum total IgE (Table 1). 

## 4. Discussion

We examined the relationship between different atopic indicators in a random sample of ~1000 children, aged 7–17 years, and identified three major classes of the studied traits relating to (1) hay fever and atopy, (2) asthma, airway responsiveness and airway obstruction, and (3) atopic dermatitis. Furthermore, we found that serum total IgE seemed to explain some cross-relation between these three groupings indicating that IgE production is an underlying trait common to the different atopic manifestations. Our analysis only retained about 55% of the variability in the studied traits consistent with a high degree of heterogeneity within the atopic syndrome. So although some categorisation could be made in regard to separating upper airway symptoms from lower airway symptoms and skin symptoms there is still a high degree of unexplained variation that cannot be sufficiently accounted for by only three latent factors. 

 Our definition of atopic diseases was based on a semistructured interview, which can be biased by parental recall and subjective interpretation. A more detailed symptom registration and longitudinal data with information on change in quality and severity of symptoms over time within the same individual could have made disease definitions more robust. Furthermore, differences in prevalence rates of atopic diseases between the two cohorts could have influenced the results. Also, inclusion of additional biomarkers, such as sputum and blood eosinophils, exhaled nitric oxide, and inflammatory proteins would have been favourable but could have induced more missing data. We tested for AHR with histamine, which lack specificity for detecting airway inflammation. Furthermore, different tests for AHR, skin test reactivity, and IgE were used in the two cohorts. Genotype data and measurements of other confounding variables such as lifestyle factors could have contributed to a more comprehensive understanding of the interrelationships between the atopic diseases. The low participation rate in the study could have led to a skewed selection of subjects; particularly there was some indication of over recruitment of symptomatic individuals, which could have had an influence on the distribution of the studied traits. Our results may only be representative for children and adolescents, whereas adults may exhibit a different pattern of correlations between traits, as would populations from other geographical areas. 

 We conclude that asthma, hay fever, and atopic dermatitis, to some extent, are characterised by different sets of biomarkers. However, a large proportion of the variation in the studied traits was not explained by our proposed decomposition indicating significant heterogeneity within the atopic syndrome. 

## Figures and Tables

**Table 1:** Correlations between atopy-related traits in a population of 1007 children, 7–17 years of age.

Trait	Traits^(1)^										Principal components^(2)^			Summary statistics^(3)^
	Asthma	Hay fever	Eczema	IgE	FEV_1_/FVC	AHR	SPT	Grass	Cat	HDM	Comp 1	Comp 2	Comp 3	
Asthma	1										024	0.67	0.12	7.1 (5.7–8.9)
Hay fever	**0.25**	1									0.66	0.10	0.17	17.3 (15.1–19.7)
Eczema	0.10	**0.14**	1								0.09	0.05	0.90	22.1 (19.6–24.7)
IgE	**0.20**	**0.32**	**0.17**	1							0.50	0.22	0.30	35.2 (31.8–39.1)
FEV**_1_**/FVC	**−0.16**	−0.05	−0.01	−0.06	1						0.04	−0.62	0.20	89.4 (89.0–89.8)
AHR	**0.38**	**0.16**	**0.13**	**0.24**	**−0.18**	1					0.11	0.74	0.22	11.6 (9.7–13.7)
SPT	**0.23**	**0.46**	**0.12**	**0.39**	−0.05	**0.20**	1				0.89	0.11	−0.05	21.8 (19.4–24.5)
Grass	**0.13**	**0.43**	0.11	**0.30**	−0.03	0.05	**0.55**	1			0.69	−0.13	0.11	7.7 (6.2–9.5)
Cat	**0.25**	**0.34**	**0.18**	**0.25**	−0.03	**0.25**	**0.50**	**0.32**	1		0.65	0.11	0.13	6.5 (5.1–8.2)
HDM	**0.24**	**0.32**	0.20	**0.30**	−0.07	**0.20**	**0.69**	**0.20**	**0.41**	1	0.71	0.24	−0.18	11.8 (10.0–14.0)

^(1)^Significant correlations are shown in bold print. Significance level is 0.0005 when adjusting for multiple testing.

^(2)^Varimax-rotated factor solution.

^(3)^IgE is geometric mean (95% CI); all other values are percentages (95% CI).

IgE is serum total IgE; AHR is airway hyperresponsiveness to histamine; SPT is positive skin prick test; HDM is house dust mite (D. Pteronyssinus).

## References

[1] Thomsen SF, Ulrik CS, Kyvik KO, Skadhauge LR, Steffensen I, Backer V (2006). Findings on the atopic triad from a Danish twin registry. *International Journal of Tuberculosis and Lung Disease*.

[2] Thomsen SF, Ferreira MAR, Kyvik KO, Fenger M, Backer V (2009). A quantitative genetic analysis of intermediate asthma phenotypes. *Allergy*.

[3] Knudsen TB, Thomsen SF, Nolte H, Backer V (2009). A population-based clinical study of allergic and non-allergic asthma. *Journal of Asthma*.

[4] Bousquet J, van Cauwenberg P, Khaltaev N, Aït-Khaled N, Annesi-Maesano I, Bachert C (2001). Allergic rhinitis and its impact on asthma. Aria workshop report. *Journal of Allergy and Clinical Immunology*.

[5] Backer V, Groth S, Dirksen A (1991). Sensitivity and specificity of the histamine challenge test for the diagnosis of asthma in an unselected sample of children and adolescents. *The European Respiratory Journal*.

[6] Thomsen SF, Ulrik CS, Larsen K, Backer V (2004). Change in prevalence of asthma in Danish children and adolescents. *Annals of Allergy, Asthma and Immunology*.

[7] Ferris BG (1978). Epidemiology standardization project. American Thoracic Society Executive Committee. *The American Review of Respiratory Disease*.

[8] Håkansson K, Thomsen SF, Ulrik CS, Porsbjerg C, Backer V (2007). Increase in the prevalence of rhinitis among Danish children from 1986 to 2001. *Pediatric Allergy and Immunology*.

[9] Stensen L, Thomsen SF, Backer V (2008). Change in prevalence of atopic dermatitis between 1986 and 2001 among children. *Allergy and Asthma Proceedings*.

[10] Cockcroft DW, Killian DN, Mellon JJA, Hargreave FE (1977). Bronchial reactivity to inhaled histamine: a method and clinical survey. *Clinical Allergy*.

[11] Yan K, Salome C, Woolcock AJ (1983). Rapid method for measurement of bronchial responsiveness. *Thorax*.

